# Study of Liver Diseases in Pregnancy and Their Implications for Maternal and Fetal Outcomes

**DOI:** 10.7759/cureus.90310

**Published:** 2025-08-17

**Authors:** Mohan B Goyal, Vijendra Kirnake, Manisha Goyal, Unnati Shende

**Affiliations:** 1 Gastroenterology and Hepatology, National Institute of Medical Science and Research (NIMS), Jaipur, IND; 2 Gastroenterology, Jawaharlal Nehru Medical College, Datta Meghe Institute of Medical Sciences (Deemed to be University), Wardha, IND; 3 Obstetrics and Gynecology, JK Lone Hospital, Jaipur, IND; 4 Obstetrics and Gynecology, Urvi Speciality Clinic and Fetal Sonography Center, Nagpur, IND

**Keywords:** cholestasis of pregnancy, fetal complications, liver diseases, maternal, pregnancy

## Abstract

Background

During pregnancy, liver disease (LD) is associated with grave consequences, for both mother and fetus, and can be a challenge for health care providers. The objective of this study is to evaluate the prevalence, the difference between common causes of pregnancy-related LD and the post-pregnancy outcomes.

Methods

The study was conducted at a tertiary care teaching hospital over 19 months. Pregnant women attending antenatal clinic with pre-existing LD or those suspected to have LD on the basis of clinical or investigation data were studied and followed up till delivery. Management was based on obstetric assessment of fetus and its well-being, cervical favorability and fetal size in combination with liver function tests.

Results

Around 126 (3.2%) of the pregnant women in the study had LD. Among them, 85 (74.5%) of the affected patients had pregnancy-specific LD and 25 (21.9%) had viral hepatitis. Out of the total 114 participants, 77 were nulliparous (67.5%) and 37 (32.5%) were multiparous. A majority (91, 79.8%) presented at >28 weeks of gestation. Intrahepatic cholestasis of pregnancy was the commonest (63, 55.3%) LD affecting pregnancy, followed by pre-eclampsia and chronic hepatitis B virus (HBV) infection 14 (12.3% each) pregnant women. There were two (1.75%) maternal deaths and five (4.2%) intrauterine deaths.

Conclusion

In pregnancies with liver disorders, the outcomes for both the mother and the fetus may be improved by early diagnosis, suitable supportive care, and a proactive policy of early delivery when indicated.

## Introduction

The human body goes through various physiological changes during pregnancy. In fact, pregnancy causes perhaps the biggest physiological stress amongst humans. So, the propensity to morbidity for both the pregnant woman and fetus is high. Liver, being the site of various important metabolic activities in the body, is naturally at risk. Liver disease (LD) in pregnancy can have serious consequences and finding the cause of LD during pregnancy can be challenging. So it is critical to make the right diagnosis to avoid morbidity or death for both the mother and the fetus.

Up to 3% of all pregnancies are reportedly complicated by liver disorders [1]. Various parameters are utilized for liver function assessment. So it is vital to appreciate the different reference ranges in pregnancy, so that the sensitivity of diagnosing liver disorders in pregnancy increases [2,3]. Typically, the serum bile acid levels are raised, while total bilirubin, alanine aminotransferase (ALT), aspartate aminotransferase (AST), and gamma-glutamyltranspeptidase (GGT) are low. In the third trimester, there is a slight increase in alkaline phosphatase. Compared to women who are not pregnant, the cholesterol level is higher, and the albumin level is lower [2-4].

In pregnancy, liver involvement is of three types: LD unique to pregnancy, LD coincidental to pregnancy, and pregnancy in patients with pre-existing LD. The LD unique to pregnancy includes hyperemesis gravidarum, acute fatty liver of pregnancy, intrahepatic cholestasis of pregnancy (ICP), and pregnancy-induced hypertension (PIH) related dysfunction, including pre-eclampsia and HELLP (hemolysis, elevated liver enzymes and low platelets) syndrome. LD such as acute viral hepatitis can occur in pregnancy and pregnancy may occur in a patient with underlying chronic LD (CLD), including patients with cirrhosis and portal hypertension and patients who have undergone liver transplantation.

A systematic research on the prevalence, etiological profile, treatment, and outcome of pregnancy-related LD in India is comparatively lacking. Thus, this present study evaluated the prevalence, the differentiation between common causes of pregnancy-related LD and the post-pregnancy outcomes.

## Materials and methods

This prevalence study was conducted by the Department of Gastroenterology and Hepatology in association with the Department of Obstetrics and Gynecology of a tertiary care teaching hospital in northern India. The study was conducted over 19 months (July 2012 to February 2014) after due approval from the institutional ethics committee. Consecutive women attending the antenatal clinic at the study hospital constituted the study population after they were confirmed to have a viable pregnancy. The confirmation of pregnancy was made by ultrasonography by crown-rump length of the fetus.

Inclusion criteria

Pregnant women attending antenatal clinic with pre-existing LD (such as metabolic dysfunction-associated steatohepatitis, viral hepatitis, and others) or those suspected to have LD on the basis of clinical or investigation data as follows were included: (a) history suggestive of LD: itching, yellowish discoloration of eye/skin/urine; (b) deranged liver function tests: serum bilirubin >2 mg/dl, AST (SGOT)/ALT (SGPT) >1.5 times upper limit of normal (>40 U/L), GGT >1.5 times upper limit of normal (>35 U/L), serum bile acid >10 umol/L; (c) positivity of viral markers such as hepatitis B surface antigen (HBsAg), hepatitis A/hepatitis E (HAV/HEV) immunoglobulin M (IgM) or anti-HCV (hepatitis C virus) antibody; (d) positivity of antibody testing for other chronic liver diseases like autoimmune hepatitis, primary biliary cirrhosis, primary sclerosing cholangitis and collagen vascular disorders; and (e) clinical evidence of portal hypertension (extrahepatic portal venous cbstruction (EHPVO), cirrhosis, non-cirrhotic portal fibrosis (NCPF)).

Exclusion criteria

Women with fetuses diagnosed with structural/chromosomal anomaly (such as triploidy, trisomy 13, trisomy 18, hydatidiform mole, fetal hydrops, severe structural cardiac malformations causing fetal compromise, and others), and those not willing to consent for the study were excluded.

Study method

The participants selected as per the inclusion criteria out of the study population were studied prospectively. Verbal and written informed consent were obtained from all women before participation in the study. All the participants underwent a comprehensive assessment. History was recorded in detail and consisted of information on demographic parameters, obstetric and medical evaluation (parity, history of complication in previous pregnancy if any, history of LD in previous pregnancy, last menstrual period, expected date of delivery, drug intake before pregnancy (oral contraceptive) and during current pregnancy, history of alcohol intake, history of pre-existing liver diseases, history of diabetes mellitus, pregnancy-induced hypertension, cardiac disease in current/previous pregnancies). The general physical and systemic examination included pulse, blood pressure, pallor, icterus, edema, ascites, sensorium, and hepatosplenomegaly.

The investigational workup consisted of liver function test (LFT), hemogram, prothrombin time (PT), international normalized ratio (INR), renal function test, etiological work-up for LD (hepatitis viral markers - HBsAg and anti-HCV - in all patients and if clinical jaundice: HAV IgM, HEV IgM antibody), ultrasound sonography (USG) abdomen (both obstetric and upper abdomen). Additional investigations to conclude the etiology of LD were undertaken in select participants wherever clinically indicated.

Operational definitions

The following clinical findings and investigations served as the basis for the diagnostic criteria for particular pregnancy LD:

Hyperemesis gravidarum with liver dysfunction - Raised bilirubin or liver enzymes in the setting of constant vomiting for more than one week during the first or second trimester [5].

Intrahepatic Cholestasis of Pregnancy (ICP) - Generalized persistent pruritus with elevated serum transaminases (>40 IU/l) and/or bile acids (fasting >10 micromoles/L) in the second or third trimester [6-8]. If it is not possible to measure serum bile acid, according to the most recent guidelines from the UK Royal College of Obstetricians and Gynecologists, a woman may be diagnosed with ICP if she exhibits standard pruritus and abnormal liver function tests, both of which subside following delivery [9].

Pre-eclamptic Liver Dysfunction - After 20 weeks of pregnancy, elevated bilirubin or liver enzymes are present along with edema, proteinuria, and hypertension [5].

Hemolysis, Elevated Liver enzymes and Low Platelet (HELLP) syndrome - HELLP syndrome was diagnosed with the presence of [10,11] elevated AST >70 IU/L, low platelet count <15,0000/L, and hemolysis (lactate dehydrogenase (LDH) >600 U/l), and elevated indirect bilirubin.

Acute Fatty Liver of Pregnancy (AFLP) - Six or more of the following symptoms, if no other explanation can be found: Abdominal pain, vomiting, ascites or bright liver on ultrasound scan, polydipsia/polyuria, encephalopathy, hypoglycemia, elevated bilirubin, elevated transaminases, elevated urate, elevated ammonia, leukocytosis, microvesicular steatosis on liver biopsy renal impairment, and coagulopathy [1,12].

Acute Viral Hepatitis (AVH) - The criteria followed were (i) new onset of jaundice without a prior history of CLD or jaundice; (ii) lack of known etiology of jaundice, including severe infections, drug hepatitis, cholestatic jaundice of pregnancy, HELLP syndrome, acute fatty liver of pregnancy; (iii) serum bilirubin level >2 mg/dL; serum alanine aminotransferase level >2.5 times the upper limit of normal; and positivity for any hepatotropic virus by following serologic tests: hepatitis B surface antigen; antibody to hepatitis C virus and IgM antibodies to hepatitis A virus, hepatitis B core antigen, hepatitis delta virus, and HEV [13]. Hepatic encephalopathy occurring within 26 weeks of the onset of AVH was considered fulminant hepatic failure.

The following additional classifications were applied to AVH: hepatitis A (IgM anti-HAV), acute hepatitis B (IgM anti-HBc and HBsAg), hepatitis C (HCV RNA and/or anti-HCV), and hepatitis E (HEV RNA IgM and/or anti-HEV).

Follow-up

All the patients were diligently followed up till delivery (outpatient department (OPD) patients - monthly, admitted patients - daily). The managing obstetricians were instructed by the study protocol to collect fasting blood samples for weekly analysis of serum bilirubin, aminotransferases, and bile acids until delivery, to request prompt contact from patients, and to use cardiotocography to monitor fetal health during the same appointments. Management was based on obstetric assessment of the fetus and its well-being, cervical favorability, and fetal size in combination with liver function tests. Acute hepatitis patients were monitored for complications. Patients with ICP received treatment with ursodeoxycholic acid (10-15 mg/kg body weight). Patients with PIH were treated with antihypertensive drugs. In women with portal hypertension, gastroesophageal varices were looked for. Patients with decompensated LD received symptomatic treatment. A first-trimester nuchal translucency scan (11-13 weeks of gestation) in patients who presented in the first and second trimesters was dome and anomaly scan (18-20 weeks) was performed in all participants for the measurement of fetal growth and examination of congenital defects. Data recorded at the delivery time included mode of delivery, gestational age, spontaneous or induced labor, asphyxial events (Apgar score and meconium staining of amniotic fluid), blood loss, and birth weight. All data pertaining to patient history, laboratory results, and delivery data were duly recorded in the patient proforma.

The outcomes were assessed and defined as follows: spontaneous preterm delivery (defined as less than 37 weeks' period of gestation), fetal growth restriction (defined as birth weight less than 10th percentile of average fetal weight for that gestation age), and low birth weight (birth weight less than 2500 grams).

Statistical analysis

Descriptive statistics were used. Continuous variables are presented as mean±SD, and categorical variables are presented as absolute numbers and percentage.

## Results

During study period of 19 months, 3829 pregnant women attended the antenatal clinic. Out of them, 126 (3.2%) pregnant women were diagnosed with liver dysfunction as per the mentioned criteria. Four women had spontaneous/missed abortion in the first trimester, two underwent medical termination of pregnancy and six had incomplete follow-up or were either lost to follow-up. Thus, 114 participants were considered for final analysis. The mean age of the participants was 27.9±5.0 years. Out of 114 participants, 77 (67.5%) were nulliparous and 37 (32.5%) were multiparous. Maximum patients (91, 79.8%) presented at >28 weeks of gestation period.

An analysis of the distribution of LD revealed ICP to be the commonest (63, 55.3%) LD affecting pregnancy, followed by pre-eclampsia and chronic HBV infection (14 (12.3%) each) (Table 1).

**Table 1:** Distribution of liver diseases in pregnancy amongst participants (n= 114)

Causes	Frequency	Percentage (%)
ICP	63	55.3
Pre-eclampsia	14	12.3
Chronic HBV	14	12.3
AVH-HEV	8	7.0
HELLP	5	4.4
CLD	3	2.6
AVH-HAV	3	2.6
AFLP	2	1.8
Hyperemesis	1	0.9
Others	1	0.9

The ICP (n=63) cases were analyzed in further detail. The mean age of those with ICP was 27.9+4.8 years, with 42 (66.7%) participants being nulliparous. The majority (55, 87.30%) of them presented after 28 weeks of gestation, with itching 51 (81%) being most common presenting complaint. The birth weight of babies of ICP patients among 63 deliveries (68 babies) was normal in 52 (76.5%) and low in 16 (23.5%, including five twins) participants. The profile of patients with ICP is presented in Table 2.

**Table 2:** Profile of patients with ICP (n=63)

Variable	Frequency	Percentage (%)
Parity		
Nullipara	42	66.7
Multipara	21	33.3
Gestation at presentation		
<12 weeks	1	1.60
13-28 weeks	7	11.10
>28 weeks	55	87.30
Presenting complaints		
Itching	51	81
Edema	6	9.5
Jaundice	6	9.5
Birth Weight (BW)		
Normal BW	52	76.5
Low BW	16 (including 5 twins)	23.5

The laboratory investigations are elaborated in Table 3.

**Table 3:** Laboratory investigations (n=63) Hb: Hemoglobin; TLC: total leukocyte count; ALT: alanine aminotransferase; AST: aspartate aminotransferase

Investigations (Mean values)	Frequency
Hb (gm%)	10.48
TLC (/mm^3^)	10.46
Platelet (x10^9^/L)	296.71
Bilirubin- total (mg/dl)	2.2
Bilirubin- direct (mg/dl)	0.299
Alkaline phosphatase (IU/L)	207.17
ALT (IU/L)	181
AST (IU/L)	148.24
Serum protein (g/dL)	7.262
Serum albumin (g/dl)	2.852
Bile acid (µmol/L)	26.11

The majority of the patients with ICP (n=63) delivered at full term (48, 76.1%), with 28 (44.4%) having had vaginal delivery and 20 (31.7%) caesarean section (LSCS). Thirteen out of 20 full-term (FT) LSCS cases were emergency sections, with failed induction of labor (seven out of 20) being the commonest indication for FT LSCS. Three out of four pre-term (PT) LSCS were emergency, with varying indications (Table 4).

**Table 4:** Mode of delivery and indications in patients with ICP ICP: Intrahepatic cholestasis of pregnancy; LSCS: caesarean section; PT: preterm; IUGR: intrauterine growth restriction; GDM: gestational diabetes mellitus.

Variable	Frequency	Percentage (%)
Mode of delivery		
Full term vaginal delivery	28	44.4
Full term LSCS	20	31.7
Preterm vaginal delivery	11	17.5
Preterm LSCS	4	6.3
Total	63	100
Mode of delivery in FT LSCS		
Emergency	13	65
Elective	7	35
Total	20	100
Indication of FT LSCS		
Failed induction of labour	7	35
Breech presentation	4	20
Cephalopelvic disproportion	2	10
Fetal distress	2	10
cervical dystocia	1	5
Non-progress of labour	1	5
Previous LSCS	1	5
Twin pregnancy in labour with first in transverse lie	1	5
Unstable lie	1	5
Total	20	100
Mode of delivery in PT LSCS		
Emergency	3	75
Elective	1	25
Total	4	100
Indication of PT LSCS delivery		
IUGR with deranged Doppler study	1	25
GDM with deranged liver enzyme	1	25
Posterior placenta previa	1	25
twin pregnancy in labour with first in transverse lie	1	25
Total	4	100

The two important hypertensive disorders of pregnancy, viz., pre-eclampsia and HELLP syndrome, were also studied in further detail. The mean age of those with pre-eclampsia (n=14) was 31.14+5.46 years and of those with HELLP syndrome (n=5) was 29.20+7.8 years, with 12 (57.1%) out of the total 19 patients with hypertensive disorders being nulliparous. The majority of patients presented after 28 weeks of gestation with pre-eclampsia (11/14, 78.57%) and HELLP syndrome (4/5, 80%), with edema feet in the women with pre-eclampsia (10/14, 71.4%) and jaundice/altered sensorium in the women with HELLP syndrome (2/5, 40%) being the most common presenting complaints. Birth weight was low in the majority of the patients (13/14 (82%), including two twins) with pre-eclampsia and normal in the majority of those with HELLP syndrome (4/5, 80%). The patient profile is detailed in Table 5.

**Table 5:** Patient profile of patients with pre-eclampsia and HELLP syndrome HELLP: Hemolysis, elevated liver enzymes and low platelets; Hb: Hemoglobin; TLC: total leukocyte count; ALT: alanine aminotransferase; AST: aspartate aminotransferase

Variable	Pre-eclampsia (n=14)	HELLP syndrome (n=5)
Parity		
Nullipara	8 (57.1%)	4 (80%)
Multipara	6 (42.9%)	1 (20%)
Gestation at presentation		
<12 weeks	0	0
13-28 weeks	3 (21.4%)	1 (20%)
>28 weeks	11 (78.6%)	4 (80%)
Presenting complaints		
Jaundice	1 (7.1%)	2 (40%)
Itching	3 (21.4%)	1 (20%)
Edema	10 (71.4%)	0
Altered sensorium	0	2 (40%)
Epigastric pain/ vomiting	5 (35.7%)	0
Previous history of PIH	1 (7.1%)	0
Investigations (Mean values)		
Hb (gm%)	10.56	8.26
TLC (/mm^3^)	9.91	10.54
Platelet (x10^9^/L)	196.7	80.2
Bilirubin- total (mg/dl)	0.803	4.1
Bilirubin- direct (mg/dl)	0.319	1.2
Alkaline Phosphatase (IU/L)	294.7	158.6
ALT (IU/L)	119.3	299.8
AST (IU/L)	99.9	213.2
Serum protein (g/dL)	5.76	6.06
Serum albumin (g/dl)	2.58	2.72
Birth weight (BW)		
Normal BW	3 (18%)	4 (80%)
Low BW	13 (82%) (including two twins)	1 (20%)

Most of the patients with pre-eclampsia delivered with LSCS (9/14, 64%), with (5/14, 36%) delivering preterm and (4/14, 28%) delivering full-term. Sixty percent of patients with HELLP syndrome delivered at full-term with LSCS. The indications of full-term LSCS in pre-eclampsia were fetal distress (n=1), PIH with failed induction (n=1), severe pre-eclampsia with toxemia (PET, n=1) and unstable lie (n=1); while non-progress of labor, severe PET, and unstable lie were the major indications in patients with HELLP syndrome. Out of five cases of preterm LSCS amongst patients with pre-eclampsia, impending eclampsia, IVF pregnancy with leaking per vaginum, polyamnios with increased diastolic flow in the middle cerebral artery with impending eclampsia, severe IUGR with oligoamnios with severe PET and severe pre-eclampsia with IUGR were the reason in one case each; while one IVF pregnancy with an operated case of ovarian cystectomy with myomectomy was the only indication for preterm LSCS in patients with HELLP syndrome.

The distribution of hepatitis amongst the study group was as follows: chronic HBV 14 (56%), AVH-HEV 8 (32%), and AVH-HAV 3 (12%). The majority of the hepatitis patients presented after 28 weeks of gestation. Jaundice and itching were the predominant symptoms in AVH-HEV and AVH-HAV patients, while those with chronic HBV infection were mostly asymptomatic. The birth weights were normal in the majority of the participants. The detailed profile is presented in Table 6.

**Table 6:** Profile of patients with Hepatitis *One died without delivery. AVH: Acute viral hepatitis; HAV: hepatitis A virus; HEV: hepatitis E virus; HBV: hepatitis B virus.

Variable	AVH-HEV (n=8)	AVH-HAV (n=3)	Chronic-HBV (n=14)
Gestation at presentation			
<12 weeks	0	1 (33.3%)	1 (7.1%)
13-28 weeks	2 (25%)	0 (0%)	3 (21.4%)
>28 weeks	6 (75%)	2 (66.7%)	10 (71.5%)
Presenting complaints			
Jaundice	8	3	0
Itching	5	1	0
Edema	1	0	0
Altered sensorium	2	0	0
Epigastric pain/ vomiting	0	0	3
Fever	3	0	0
Asymptomatic	0	0	11
Birth Weight			
Normal BW	3 (37.8%)	2 (66.7%)	12 (85%)
Low BW	4 (50%)	1 (33.3%)	2 (15%)
Total	7*	3	14
Mode of delivery			
Full term vaginal delivery	3 (37.5%)	1 (33.3%)	7 (50%)
Preterm vaginal delivery	1 (12.5%)	0	2 (14.3%)
Full term LSCS	1 (12.5%)	1 (33.3%)	3 (21.4)
Preterm LSCS	2 (25%)	1 (33.3%)	2 (14.3%)

There were a total of two (1.75%) maternal deaths and five (4.2%) intrauterine deaths (IUD) during the study. The details of the maternal and perinatal deaths are summarized in Table 7.

**Table 7:** Maternal and perinatal death HEV: hepatitis E virus; HBV: hepatitis B virus; HAV: hepatitis A virus; AFLP: acute fatty liver of pregnancy; HELLP: hemolysis, elevated liver enzymes and low platelets; CLD: chronic liver disease

Disease (n)	Maternal deaths (%)	Perinatal deaths (%)
HEV (8)	1 (12.5%)	1 (12.5%)
HAV (3)	0	0
HBV (14)	0	0
AFLP (2)	1 (50%)	2 (100%)
HELLP (5)	0	0
Cholestasis (63)	0	1 (1.58%)
Pre-eclampsia (14)	0	0
Hyperemesis (1)	0	0
CLD (3)	0	1(33.3%)
Any Other (1)	0	0
	2/114 women (1.75%)	5/119 fetuses (4.2%)

## Discussion

Deranged liver function parameters during pregnancy is a commonly encountered problem. It is important to study LD in pregnancy comprehensively, along with an analysis of its implications for maternal and fetal outcomes. As a northern Indian focused study on the entire spectrum of LD in pregnancy was lacking, this particular study was planned to fulfil the lacuna. Women with pre-existing LD or those clinically suspected to have LD were evaluated with hepatitis viral markers, liver biochemistry, hepatobiliary sonography, and, when required, further investigations to identify the causes of LD along with a thorough clinical assessment with clinical follow-up till delivery to evaluate outcome.

A total of 126 (3.2%) out of 3829 pregnant women were detected with liver dysfunction, out of which 114 women completed follow-up. The incidence is similar to findings of Ch’ng et al., who reported live dysfunction in 3% of a total of 4377 deliveries during their 15-month-long study in Southwest Wales [1]. Another study by Hay observed abnormal liver parameters in 3%-5% pregnancies [14]. The results are in contrast with a prospective study from southern India, with 0.34% of 36,486 pregnant women having a LD, while another Indian study reported the incidence at 0.9% [15,16]. The reason for the relatively high incidence in the present study could be referral bias, with the study center being a tertiary care hospital with comparatively better nursery care.

In the present study, 85 (74.5%) patients had pregnancy-specific LD and 25 (21.9%) had viral hepatitis. The reported proportion of pregnancy-specific LD among affected patients in previous studies varied from 67% to 89% [1,17]. A study by Rathi et al. found 56 (52%) patients with pregnancy-specific LD and 24 (24%) having viral hepatitis [16]. ICP was observed to be the commonest (63, 55.3%) LD affecting pregnancy, present in 1.6% (63/3829) of all pregnancies. The incidence of ICP ranges from 0.2% to 2% but reportedly varies widely based on geographic location and ethnicity [18]. The prevalence in Europe, United States, Canada, and Australia was reported at 0.1% to 1.5%, respectively, lower than what was observed in the present study [19-21]. In our ICP group, 55 (87.3%) women presented in the third trimester, which is the usual occurrence [22,23]. Although ICP usually commences in the third trimester, earlier start has been reported. However, there is no proof that women who seek aid earlier have worse perinatal outcomes or more severe disease. The presenting feature of ICP is usually pruritus, which starts in the palm and soles and resolves postpartum, as was evident in the present study. Clinical jaundice is routinely detected in only around 10% of the cases only with bilirubin levels rarely exceeding 100 μmol/L; its diagnosis is confirmed by raised liver transaminases and/or serum bile acids [8,19]. When elevated in a woman with typical pruritus, serum bile acid is the most reliable diagnostic marker for ICP in the absence of evidence for an alternative diagnosis [24]. As was expected, all the 63 (55.3%) women in the present study diagnosed with ICP had elevated fasting bile acid (>10 umol/L) and elevated ALT and AST levels. All the women with ICP were treated with ursodeoxycholic acid (10-15 mg/kg body weight) and all of them reported significant relief from itching. Ursodeoxycholic acid was evaluated in a largest randomized controlled trial and demonstrated similar significant reduction in pruritus, and the mechanism of action is still not fully understood though [25]. A total of 39 (61%) patients were delivered by normal vaginal delivery (28 full term, 11 preterm) and 24 (39%) were delivered by LSCS (20 full term, four preterm). Elective LSCS was done in eight cases, with preterm LSCS being done in four cases. There are currently no guidelines on the elective early delivery of ICP from the American College of Obstetricians and Gynecologists. Although there is no evidence to support or contradict this practice, many clinicians have adopted it, according to the current Royal College of Obstetricians and Gynecologists guidelines for ICP. Iatrogenic preterm delivery rates were 17%, according to a recent population-based study conducted in the United Kingdom. There was no proof that a higher rate of emergency cesarean deliveries was caused by the fact that most early deliveries were induced [26]. A study by Glanz et al. showed that the risk of fetal complications was comparable between women with ICP and a general obstetric population. In comparison to women with normal bile acid levels and those with mild ICP, 19% of women with a severe form of ICP and bile acid levels greater than 40 umol/L had a significantly higher rate of fetal complications [27]. We observed that maternal and fetal outcomes were not affected by ICP.

In the present study, 19 (16.7%) cases presented with PIH-related liver dysfunction, including 14 (12%) with pre-eclamptic liver dysfunction and five (4.4%) with HELLP syndrome. The incidence of preeclampsia is similar to the observations by Rathi et al. [16]. The mode of delivery and respective indication in pre-eclamptic cases were also in line with previous similar studies. It was previously observed that pre-eclampsia increases the rate of caesarian delivery, preterm labor, and low birth weight babies. No maternal or fetal mortality was observed amongst the pre-eclamptic group.

The incidence of HELLP is also in line with previous similar studies [10,11,23]. Although it can happen at any age or parity, HELLP is more common in older, white, and multiparous patients [28]. In our study, four women were nulliparous and one was multiparous, with the mean age of 29.20+7.8 years. Out of the five (4.4%) women with HELLP, one (20%) had normal vaginal delivery and four (80%) delivered by LSCS (elective two, emergency two). Four (80%) women delivered low birth weight babies and one (20%) delivered normal birth weight baby. Although no maternal and perinatal mortality was reported, increased LSCS rate and low birth weight babies were observed.

Although not very common, preeclampsia and HELLP syndrome remain a significant cause of morbidity and mortality for both pregnant women and their fetuses, with a reported incidence of maternal mortality of 1% in severe preeclampsia, up to 5% in HELLP syndrome, and up to 30% fetal mortality in one study [28,29]. Maternal and perinatal mortality was not seen with PIH-associated liver dysfunction in the present study. The reasons for this improvement in mortality are likely to be early recognition and treatment by prompt delivery and appropriate intensive care management.

Viral hepatitis (VH) in pregnancy has been a matter of considerable debate. Studies from the West have shown that AVH has no special predilection to pregnancy and the disease has a similar clinical course to that in non-pregnant women [30]. In contrast, viral hepatitis in developing countries has increased incidence and severity in pregnancy, which could be related to varying etiologies of AVH and fulminant hepatic failure (FHF) in developed and developing countries [31]. In the present study, 25 (21.9%) women were found to have met the diagnostic criteria of viral hepatitis. Out of them, eight (32%) were diagnosed with acute HEV infection, three (12%) with acute HAV infection, and 14 (56%) with chronic HBV infection. No acute cases of HBV and HCV were found. The HEV infection has been responsible in most of the studies, but the proportion may vary due to the small samples and regional differences [32,33]. Pregnant women with HEV in highly endemic areas are particularly at risk with up to 60% developing FHF with maternal death rate reported up to 31% of the cases [31-33]. Two cases (25%) had FHF on admission in the present study, one of which succumbed due to late presentation despite all efforts. AFLP was significantly associated with fetal (100%) and maternal (50%) mortality. In our study, four (50%) HEV cases delivered by normal vaginal delivery (full term three, preterm one) and three (37.5%) by LSCS (full term one, preterm two). One case died without delivery. Three babies had normal birth weight while four had low birth weight. Patra et al. also reported a significantly higher proportion of preterm deliveries in HEV-infected women [13].

We encountered three (2.6%) pregnant women with CLD, including one (33.3%) with Budd-Chiari syndrome and two (66.7%) with cryptogenic cirrhosis. All of them had portal hypertension and two presented with gastrointestinal bleed only. One patient had intrauterine fetal death as an outcome.

This study had certain limitations. First, it was single-center study, so multicentric studies are required to support our findings. Second, the study site was a tertiary advanced hospital in a metropolitan city, so it may not represent the prevalence of LD in rural pregnant populations. Moreover, the study may have referral bias as our institute is a tertiary referral center.

## Conclusions

In the current era of obstetrics, there is still much uncertainty regarding the best way to treat pregnancy with cirrhosis and portal hypertension. All pregnant women with liver diseases should be monitored by a hepatologist and a high-risk obstetrician. Even if there were no varices prior to pregnancy, all patients should have an upper endoscopy to check for them in the second trimester. If they do, non-selective beta blocker therapy, endoscopic ligation, and/or sclerotherapy should be started. The outcomes for both the mother and the fetus in pregnancies with LD may be improved by an early diagnosis, high index of suspicion of LD, timely referral to a higher center when necessary, appropriate supportive management, and a proactive policy of early delivery when indicated.
